# Optimal transport reveals immune perturbation and fingerprints over time in COVID-19 vaccination

**DOI:** 10.3389/ebm.2025.10445

**Published:** 2025-05-21

**Authors:** Zexuan Wang, Jiong Chen, Matei Ionita, Qipeng Zhan, Zhuoping Zhou, Li Shen

**Affiliations:** ^1^ Graduate Group in Applied Mathematics and Computational Science, University of Pennsylvania, Philadelphia, PA, United States; ^2^ Department of Bioengineering, University of Pennsylvania, Philadelphia, PA, United States; ^3^ Institute for Immunology and Immune Health, University of Pennsylvania Perelman School of Medicine, Philadelphia, PA, United States; ^4^ Department of Biostatistics, Epidemiology and Informatics, University of Pennsylvania Perelman School of Medicine, Philadelphia, PA, United States

**Keywords:** optimal transport, COVID-19 vaccination, immunity, mass cytometry, fingerprint

## Abstract

Mass cytometry enables high-throughput characterization of heterogeneous cell populations at single-cell resolution, using metal isotopes to capture cellular signals and avoiding the spectral overlap common in flow cytometry. Despite advancements, conventional data analysis often focuses on manual gating or clustering within specific samples, overlooking disparities across subjects or biological samples. To address this gap, we propose a novel framework that treats the cell-by-protein matrix as a high-dimensional distribution, using Quantized Optimal Transport (QOT) to quantify distances between samples based on their cellular protein expression profiles. This approach allows for a direct comparison of distributions without relying on predefined gating strategies, capturing subtle variations in the data. We validated our method through two experiments using real-world time-series Coronavirus Disease 2019 (COVID-19) cytometry data. First, we conducted a leave-one-out analysis to identify immunologically unstable proteins over time, revealing CD3 and CD45 as the proteins changing the most during the vaccine response. Second, we aimed to capture individual immune fingerprints over time by calculating pairwise Wasserstein distances between samples and applying hierarchical clustering. Using silhouette scores to evaluate clustering effectiveness, we identified optimal combinations of immunological markers that effectively grouped samples from the same participant across different time points. Our findings demonstrate that the QOT framework provides a robust and flexible tool for cohort-level analysis of mass cytometry data, enabling the identification of unstable immunological markers and capturing immune response heterogeneity among vaccinated cohorts.

## Impact statement

Mass cytometry enables high-throughput characterization of cellular heterogeneity, but conventional analysis often focuses on manual gating or clustering within specific samples. We propose a novel quantitative framework that directly compares the high-dimensional protein expression distributions between samples using Quantized Optimal Transport. This approach captures subtle differences without relying on predefined gating strategies. Experiments on real-world COVID-19 cytometry data identified CD3 and CD45 as the most unstable proteins during the vaccine response. Furthermore, by calculating pairwise distances and applying hierarchical clustering, we determined optimal protein combinations that effectively grouped samples from the same individual over time, reflecting unique immune fingerprints. Our findings showcase the power of this framework for cohort-level mass cytometry analysis, enabling the discovery of key immunological changes and individual response patterns.

## Introduction

Mass Cytometry (Cytometry by Time-Of-Flight) is a high-throughput technology to characterize heterogeneous cell populations in a single cell resolution [1]. As an advancement over traditional flow cytometry, mass cytometry utilizes isotopes instead of fluorophores to capture cellular signals, making a broader range of features available and avoiding the experimental difficulties related to spectral overlap [2]. In comparison with conventional single-cell RNA-seq experiments, mass cytometry also provides a higher throughput, which is capable of handling millions of cells along with a lower dimension of the cellular features derived from surface antigens, thus allowing more accurate capture of precise cell subpopulations [3]. Moreover, mass cytometry uses antibodies labeled with elemental heavy metal ions via chelating polymers to measure target proteins on single cells directly. In this method, stained cells are nebulized, vaporized, and ionized; the resulting ion cloud is mass-filtered to remove low-mass ions and then analyzed by time-of-flight mass spectrometry, precisely quantifying bound antibodies and revealing the expression of markers of interest, making it an ideal technique for monitoring the human immune system [4, 5]. The primary data analysis of mass cytometry experiments usually involves either manually separating cell subpopulations on a bivariate setting where the process is referred to as “manual gating” [6], supervised cell annotation trained by manual label [7–9], or via unsupervised clustering algorithms to group cells together [10–12]. However, these approaches often do not learn the disparities across subjects or biological samples, but they try to interpret the relationships of cells within a specific sample. Comparing the mass cytometry profile in a systematic resolution will also provide benefits in investigating global variation and differences [13, 14]. Characterizing and tracing the entire cell population would not only enable a more comprehensive understanding of how immune response varies systematically but also differentiate between samples and various cell subtypes in different diseases [15–18].

Optimal Transport (OT) is a mathematical framework originally proposed by Monge [19] and later reformulated by Kantorovich into a computationally tractable form [20]. OT addresses the challenge of comparing empirical distributions by finding the most efficient way to transform one distribution into another, ensuring mass preservation while minimizing an associated cost function. Recently, OT has been applied to mass cytometry data for automatic gating [21, 22].

**FIGURE 1:**
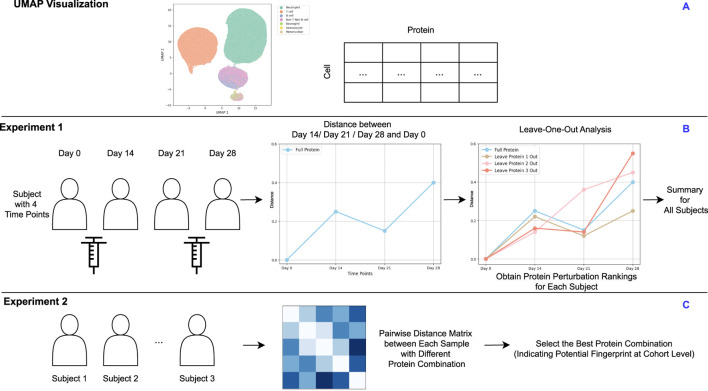
**(A)** UMAP visualization of a representative sample. **(B)** Experiment 1: For subjects measured at multiple time points, proteins are ranked by their level of perturbation within each subject. These rankings are then aggregated to identify proteins showing the greatest instability across the cohort. **(C)** Experiment 2: Each subject measurement at a specific time point is treated as an individual sample. Pairwise distance matrices are computed using various protein combinations to identify the optimal combination that effectively clusters samples. This optimal protein combination reflects the cohort-level fingerprint. **(A)** is based on real data, while **(B,C)** are derived from a synthetic design.

Despite numerous algorithms developed for manual gating, practical methods for downstream analysis are still lacking. Traditional studies often compare disease states by focusing on the proportions of gated cell populations among cohorts to infer protein importance and disease-related protein expression [5, 23]. This approach may overlook differences in protein expression levels within cell populations. This work proposes a novel framework that treats the cell-by-protein matrix as a high-dimensional distribution, with each protein representing a dimension. By representing each sample as a distribution of cells across these protein dimensions, we can directly compare the distributions between cohorts using Optimal Transport. This allows us to quantify differences in protein expression profiles without relying on predefined gating strategies, capturing more nuanced variations in the data. Our main contributions are:

1. Quantifying Subject Differences via Quantized Optimal Transport: We introduce a method that utilizes Quantized Optimal Transport (QOT) to quantify the distance between subjects, viewing each cohort as a distribution of cells in high-dimensional protein expression space. This strategy can be applied with or without prior gating, providing flexibility in analysis.
2. Demonstrating Effectiveness on Coronavirus Disease 2019 (COVID-19) Cytometry Data: We validate our method through two experiments using real-world time-series COVID-19 cytometry data (Figure 1A). Specifically, we focus on (i) identifying immunologically unstable proteins over time (Figure 1B) and (ii) identifying informative proteins that contribute to fingerprint differentiation (Figure 1C). These case studies highlight the utility of our approach in revealing immune stability and heterogeneity of immune responses among vaccinated cohort.

## Materials and methods

We evaluated our approach on a synthetic dataset—with three cohorts, each sampled at three-time points—and a real-world mass cytometry dataset of single-cell protein expression in immune cells [24]. Additionally, in this paper, the term “cohort” refers to the entire dataset under study. To avoid confusion, we use the term “subject” to denote all time points belonging to the same individual. The term “sample” is used to refer to an individual file within the dataset, representing a specific time point for a individual.

**FIGURE 2:**
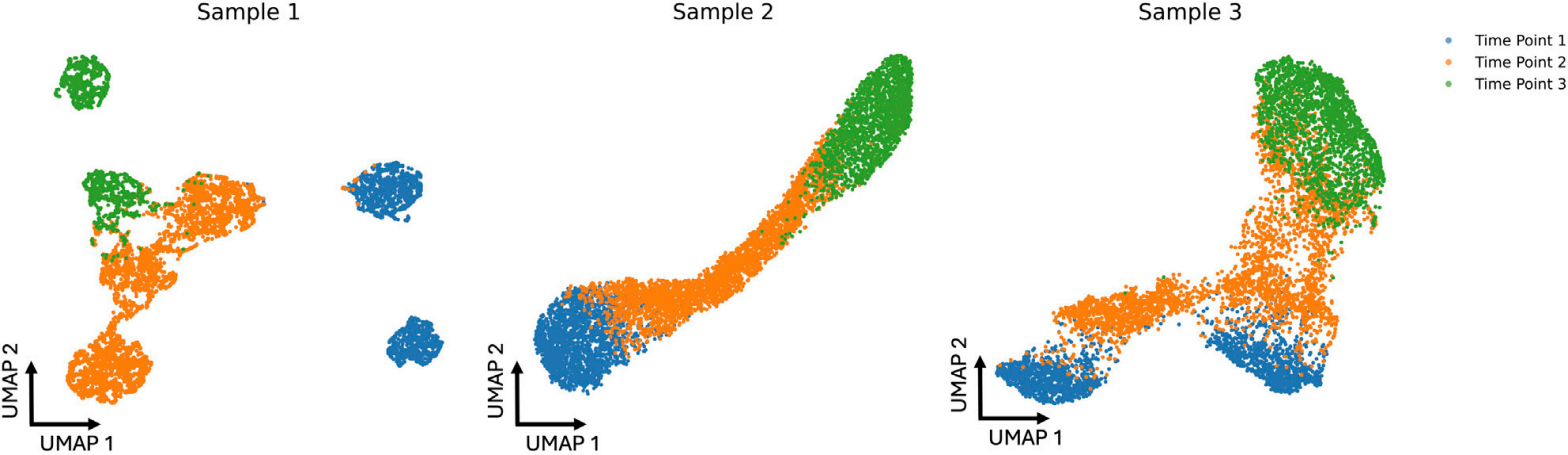
UMAP projections of three simulated cohorts at three different time points (blue, orange, and green). Each sample’s distribution reveals distinct cell patterns that change over time, showing the dynamic shifts in the data across the simulated time course.

In our synthetic dataset, we introduced distinct evolutionary patterns to capture heterogeneity within each subject. Specifically, Subject 1 follows a branched trajectory, with separate cell populations diverging from Time 1 to Time 2, and again from Time 2 to Time 3. Subject 2 evolves along a smooth, curved progression, while Subject 3 exhibits a Y-shaped branching pattern, where all cells transition to new states from a common lineage. Each subject is characterized by 2, 3, and 2 cell types at Time 1, Time 2, and Time 3, respectively. Subject 1 has disproportionately sized cell types but a total of 7,000 cells across all time points. By contrast, Subjects 2 and 3 each maintain 1,000 cells per cell type, also yielding 7,000 cells in total. Further details on the cell-type proportions for Subject 1 can be found in Supplementary Appendix Table S1. The UMAP projection of the cohorts is shown in Figure 2.

For the real-word datasets, Whole blood was profiled from a cohort of 37 healthy subjects at multiple time points during two-dose mRNA vaccination against SARS-CoV-2. Each sample contains approximately 321 k cells. Most blood draws occurred at four standardized time points: a baseline draw before the first dose (T1), 2 weeks after the first dose (T2), before the second dose (T3), and a week after the second dose (T4). A few subjects had extra blood draws between T1 and T4 at intermediate time points. This yielded a total of 150 blood samples since not all subjects were available for each time point. The whole blood samples were stained with the Maxpar Direct Immunophenotyping Assay, a standardized panel for broad immunophenotyping of immune cell types. Finally, data was collected on a CyTOF2 instrument. Demographic and vaccination details is shown in Table S.2, S.3, S.4, S.5.

### Quantized optimal transport

In this section, we briefly explain the Quantized Optimal Transport (QOT) method [25] for calculating distances at the sample level based on high-dimensional mass cytometry data.

Given a collection of P samples, denoted as $G=\left\{G_{1},G_{2},\ldots ,G_{P}\right\}$ , each sample $G_{k}$ is represented by an $n_{k}\times m$ matrix, where $n_{k}$ is the number of cells in sample k , and m is the number of features (proteins). Our framework aims to compute the distance between two samples based on their cellular protein expression profiles.

We first model each sample as a distribution defined by its protein expression levels to compute the distance between two samples. This involves two main steps: (1) fitting a Gaussian mixture model (GMM) to each sample’s data (Equations 1, 2) and (2) calculating the distance between the samples using their corresponding GMMs (Equations 3-9). For simplicity, we will use GMM as a short abbreviation for the Gaussian mixture model throughout the rest of this manuscript.

Each sample $G_{k}$ is modeled as a GMM:

$$\omega_{k}=\overset{H_{k}}{\underset{h=1}{\sum }}\alpha_{k,h}N\left(\mu_{k,h},\Sigma_{k,h}\right),$$ (1)

where $H_{k}$ is the number of Gaussian components in the GMM for sample k , $\alpha_{k,h}$ are the mixture weights satisfying

$$\overset{H_{k}}{\underset{h=1}{\sum }}\alpha_{k,h}=1\;\text{and}\;\alpha_{k,h}\geq 0,$$ (2)

$\mu_{k,h}\in R^{m}$ are the mean vectors, and $\Sigma_{k,h}\in R^{m\times m}$ are the covariance matrices of the Gaussian components. This approach allows the GMM to effectively encapsulate the distribution of the high-dimensional cytometry data for each sample.

Distances between cohorts are computed using the Wasserstein distance, quantifying the minimal cost of transporting one probability distribution into another. Specifically, we compute the Wasserstein distance between the GMMs representing the samples.

The distance between two samples, represented by their respective GMMs $\omega_{i}$ and $\omega_{j}$ , is computed by solving the following optimal transport problem:

$$\underset{T\in \underset{H_{i}\times H_{j}}{R}}{\min}\overset{H_{i}}{\underset{p=1}{\sum }}\overset{H_{j}}{\underset{q=1}{\sum }}T_{pq}C_{pq},$$ (3)

subject to the constraints:

$$\overset{H_{j}}{\underset{q=1}{\sum }}T_{pq}=\alpha_{i,p},\;\forall p=1,\ldots ,H_{i},$$ (4)

$$\overset{H_{i}}{\underset{p=1}{\sum }}T_{pq}=\alpha_{j,q},\;\forall q=1,\ldots ,H_{j},$$ (5)

$$T_{pq}\geq 0,\;\forall p=1,\ldots ,H_{i};\;\forall q=1,\ldots ,H_{j},$$ (6)

where $T_{pq}$ represents the amount of mass transported from the p -th Gaussian component of $\omega_{i}$ to the q -th Gaussian component of $\omega_{j}$ , and $C_{pq}$ is the cost of transporting unit mass between these components.

The cost matrix $C\in R^{H_{i}\times H_{j}}$ has entries defined as:

$$C_{pq}=W_{2}^{2}\left(N\left(\mu_{i,p},\Sigma_{i,p}\right),N\left(\mu_{j,q},\Sigma_{j,q}\right)\right),$$ (7)

where $W_{2}^{2}$ denotes the squared Wasserstein distance between two Gaussian distributions. The squared Wasserstein distance between the Gaussian components is given by:

$$\begin{array}{c} W_{2}^{2}\left(N\left(\mu_{i,p},\Sigma_{i,p}\right),N\left(\mu_{j,q},\Sigma_{j,q}\right)\right)=\|\mu_{i,p}-\mu_{j,q}{\|}^{2}+\mathrm{Tr}\left(\Sigma_{i,p}+\Sigma_{j,q}-2{\left(\Sigma_{i,p}^{1/2}\Sigma_{j,q}\Sigma_{i,p}^{1/2}\right)}^{1/2}\right), \end{array}$$ (8)

where ‖⋅‖ denotes the Euclidean norm, Tr(⋅) is the trace operator, and $\Sigma^{1/2}$ denotes the matrix square root of Σ . An alternative approach is to consider GMMs as point clouds instead of distribution, which provides scalability for larger-scale datasets. This approach involves calculating the cost matrix using the cosine distance between the centroids of Gaussian Mixture Models (GMMs):

$$C\left(p,q\right)=1-\frac{\mu_{i,p}\cdot \mu_{j,q}}{|\mu_{i,p}|2|\mu j,q{|}_{2}}\;$$ (9)

### Experimental designs

#### Stability of cohorts across time

To identify immunologically unstable proteins across the subjects, we conducted a leave-one-out analysis to determine which proteins, when excluded, would result in the most perturbation in the immune profiles over time. This approach allowed us to assess the stability of each protein by measuring its impact on the temporal distributional similarity of immune profiles.

For each time point measurement within each subject, we first calculated the Wasserstein distance between the baseline time point (T1) and each subsequent time point (T2, T3, T4) using the full set of immunological proteins. This provided a reference measure of distributional change with all proteins included over time. Mathematically, the Wasserstein distance $D_{t}^{\text{full }}$ between T1 and time point t (where t∈{ T2, T3, T4} ) was calculated as:

$$D_{t}^{\text{full }}=\mathrm{Dist}\left(G_{\text{T}1},G_{t}\right)$$

where Dist denotes the QOT distance calculated in previous section, and $G_{\text{T}1}$ and $G_{t}$ represent the protein distribution profiles at T1 and time point t , respectively.

We then systematically excluded one protein at a time from the dataset. After excluding a protein, we recalculated the Wasserstein distances between T1 and each subsequent time point for each participant, obtaining $D_{t}^{\text{excl }}$ . This process was repeated for every protein in the dataset, resulting in a set of perturbed Wasserstein distances corresponding to each excluded protein.

To quantify the perturbation caused by the exclusion of each protein, we calculated the absolute difference between the Wasserstein distances with all proteins included and with one protein excluded for each time point:

$$\Delta D_{t}=\left|D_{t}^{\text{full }}-D_{t}^{\text{excl }}\right|$$

We then summed these absolute differences across all time points to obtain a total perturbation score for each protein:

$$\Delta D_{\text{total }}=\underset{t}{\sum }\Delta D_{t}=\underset{t}{\sum }\left|D_{t}^{\text{full }}-D_{t}^{\text{excl }}\right|$$

a larger $\Delta D_{\text{total }}$ indicated that the excluded protein had a significant impact on the temporal stability of the immune profile, suggesting it is unstable protein over time.

We ranked the proteins for each subject based on the magnitude of $\Delta D_{\text{total }}$ their exclusion caused, from the least to the most perturbing. By aggregating these rankings across all subjects, we identified proteins that consistently resulted in the most perturbation when excluded. Proteins frequently ranked as causing the maximum perturbation across subjects were considered the most immunologically unstable over time.

#### Finger print of cohorts

In addition to identifying stable immunological features, we conducted a second experiment to capture subject immune fingerprints over time. The goal was to determine the optimal combination of immunological markers to effectively cluster samples from the same participant across different time points despite natural variations due to vaccination or immune fluctuations.

To achieve this, we calculated pairwise Wasserstein distances between all samples based on their immunological marker distributions, providing a quantitative measure of dissimilarity between samples. We then evaluate the effectiveness of different combinations of immunological protein expression with the silhouette score. The silhouette score assesses how well each sample fits within its assigned cluster compared to other clusters, offering a metric for the quality of the clustering solution. By testing various combinations of immunological protein expression and calculating the corresponding silhouette scores, we identified the feature sets that most effectively clustered samples from the same subject. In addition, we employed a UMAP visualization in which samples are connected if they meet two criteria: (1) they belong to the same group, and (2) their Euclidean distance is below a specified threshold (0.8).

## Results

### Cohort-level analysis of simulation dataset

**FIGURE 3:**
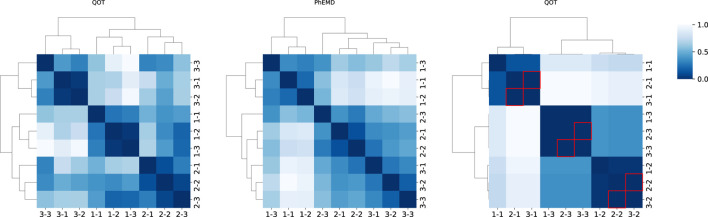
Heatmaps of pairwise distance matrices for three cohorts, computed by three methods (QOT, PhEMD, and PILOT). The label “1-1” indicates cohort 1 at time point 1. Each heatmap is hierarchically clustered, with darker shades signifying higher distances. In the PILOT panel, red boxes highlight entries where the distance is zero (excluding the diagonal).

In the cohort analysis of our simulation dataset, we compared QOT with two state-of-the-art approaches, PhEMD and PILOT, and examined their respective cohort-level distance matrices (Figure 3). Ideally, a well-structured distance matrix should exhibit a block diagonal pattern, where each block represents the same subject measured at different time points. Both QOT and PhEMD reveal these per-subject relationships clearly. In contrast, PILOT produces a mixed pattern: its hierarchical clustering intermingles different subjects, indicating it does not preserve the per-cohort structure. Moreover, PILOT assigns zero distances (highlighted by red boxes) for certain entries, suggesting identical samples. This misleading result arises from the methodology of PILOT. Specifically, PILOT first creates a uniform mask across all subjects and then considers only the proportions of cell types when computing pairwise distances. As a result, if two samples (e.g., Cohort2, Time1 and Cohort3, Time1) both contain the same set of cell types in identical proportions, PILOT assigns a zero distance, even if their expression levels differ substantially. Consequently, the uniform mask obscures critical differences in the data, failing to capture the true biological variability.

We quantitatively evaluated each distance matrix using the Silhouette score, Adjusted Rand Index (ARI), and runtime, as shown in Table 1. The Silhouette score assesses how well each sample is grouped within its own cluster and separated from others, while the ARI quantifies the agreement between true and predicted cluster assignments (with 1.0 indicating perfect alignment). Both QOT and PhEMD correctly distinguish different cohorts, achieving an ARI of 1.0. However, QOT produces a more pronounced cluster structure, reflected in a higher Silhouette score. In terms of computational efficiency, QOT completes in 5.26 s, compared to PhEMD’s 1,440 s, demonstrating superior scalability for large-scale analyses. By contrast, PILOT fails to cluster cohorts correctly, often yielding misleading zero distances and not preserving the expected block-diagonal structure.

**TABLE 1:** Performance comparison of QOT, PhEMD, and PULOT based on the Silhouette Score, Adjusted Rand Index (ARI), and Runtime.

Method	Silhouette score	ARI	Runtime (s)
QOT	0.529	1.00	5.26
PhEMD	0.429	1.00	1,440
PILOT	−0.340	−0.333	1.20

### Cohort-level analysis of COVID-19 reveals immunologically unstable protein

**FIGURE 4:**
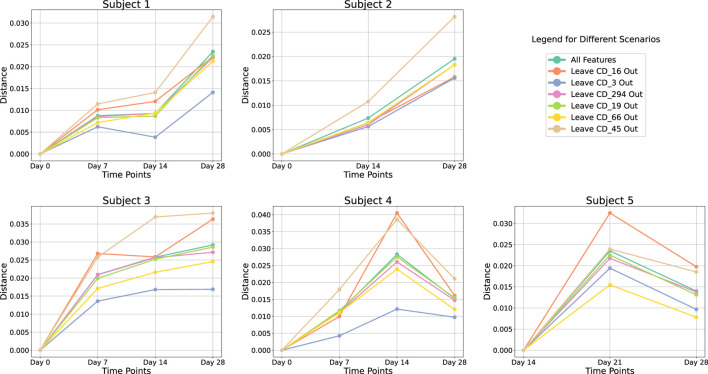
Leave-One-Out analysis at subject-level. Each line represents the perturbation in distance relative to the baseline at Day 0 over various time points. Different colored lines indicate the results using all proteins *versus* excluding a specific protein.

**FIGURE 5:**
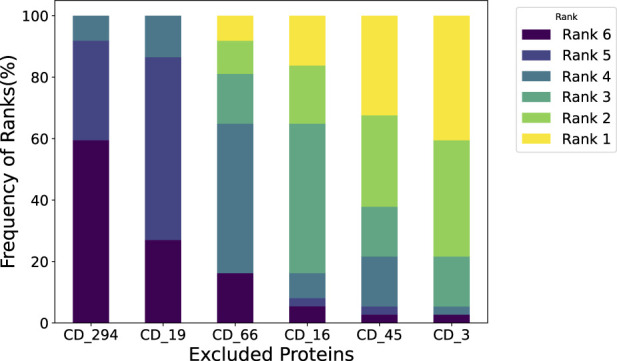
Leave-One-Out analysis at cohort-level. We assessed the impact of the subject when excluding each immunological protein and then summarized it at the cohort level. For instance, a 30% frequency at Rank 1 for CD 3 indicates that when measuring perturbation, perturbation caused by removing CD 3 ranks first in 30% of the subjects.

In our cohort-level analysis of COVID-19, we aimed to identify immunologically unstable proteins across 37 healthy subjects. We employed a leave-one-out (LOO) approach, systematically excluding each protein to evaluate its contribution to immune perturbations over time. Figure 4 illustrates the subject-level LOO results, where each line traces the distance of a subject’s sample at Day 7, 14, or 21 from its baseline (Day 0) under two conditions: using all available features *versus* excluding a specific protein. The horizontal gap between these lines shows how strongly the excluded protein influences the observed perturbation. For instance, if removing CD16 produces a significant shift in distance relative to baseline, it implies that CD16 is a key driver of the subject’s immune response over time; conversely, a negligible gap suggests that removing a protein has minimal effect and is more stable. Complete subject-level analyses are provided in the (Supplementary Appendix Figure SA1–SA3). From these LOO assessments, we found that subjects 1 through 4 showed CD3 and CD45 as their most unstable proteins, whereas subject 5’s data highlighted CD16 and CD66 as the most variable over time. We then aggregated these subject-level findings to derive cohort-level insights, presented in Figure 5. Consistently, CD3 emerged as the most unstable protein across the overall cohort, followed closely by CD45.

Furthermore, our analysis indicates that removing CD45 leads to a higher distance from baseline. In other words, when CD45 is present, it helps keep the measured distance lower, suggesting a regulatory or stabilizing role. This finding aligns with the work of Hermiston et al., who showed that CD45 modulates signals from integrins and cytokine receptors [26], as well as Priest et al., who reported that CD45 expression on B cells shapes functional memory subsets post-vaccination [27]. By contrast, removing CD3 causes the distance from baseline to decrease, implying that including CD3 consistently drives the distance upward. This indicates that CD3 is a more perturbed protein in our dataset. Supporting this observation, Sattler et al. found that following SARS-CoV-2 vaccination, high-avidity spike-specific CD4 T cells lost surface CD3 expression after *in vitro* antigen restimulation, reflecting dynamic changes in T cell activation [28]. Similarly, Jaber et al. documented heightened CD3 T-helper cell responses in COVID-19 vaccine recipients [29], underscoring the pivotal role of CD3 in mediating immune perturbations in this setting.

### Immune biomarkers for temporal fingerprint clustering

**FIGURE 6:**
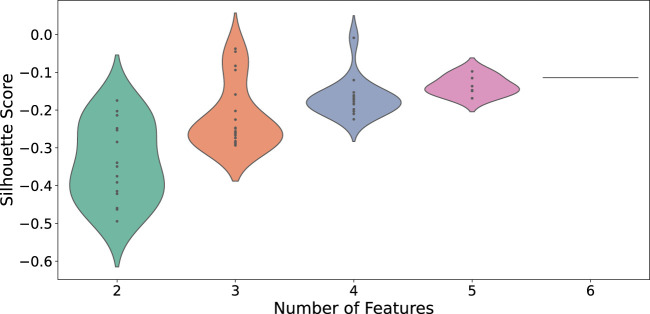
Distribution of silhouette scores across different feature counts for CD protein combina-tions. Each violin’s width represents the density of silhouette scores for that feature count.

**FIGURE 7:**
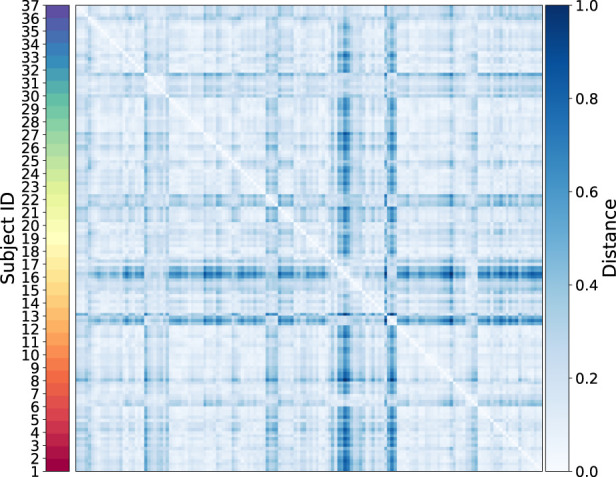
Heatmap of the sample-level distance matrix. Distance values are color-coded, with lighter shades of blue indicating closer proximity and darker shades representing greater distances. The color bar on the right provides the distance scale, while a second color bar on the left annotates subject IDs.

To identify Temporal Fingerprint Clusters across subjects, we treated each visit (timepoint) as an individual sample. Consequently, data from 37 healthy subjects resulted in 147 total samples for this analysis. Our working hypothesis is that, in an ideal scenario, samples originating from the same subject would naturally cluster together, reflecting each individual’s inherent characteristics. We then evaluated combinations of proteins to determine which set yields the most informative clustering, as shown in Figure 6. We find combination of CD19, CD16, CD294, CD66b yiels highest silhouette score. We calculated distance matrices using subsets of these proteins—ranging from two to six proteins per subset. The most effective protein combination results, as indicated by the highest silhouette score, are illustrated in Figure 7. For visualization, we employed UMAP to project the distance matrix corresponding to the optimal silhouette score.

**FIGURE 8:**
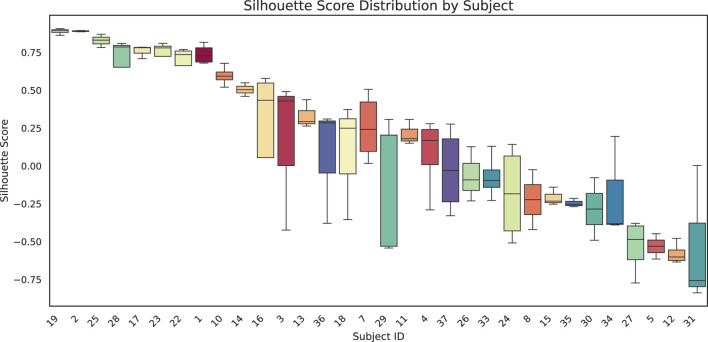
Box-and-whisker plots show the distribution of silhouette scores for each subject. Each box represents the interquartile range for that subject, with the black horizontal line indicating the median silhouette score. Higher (positive) values suggest more cohesive clustering, whereas lower (or negative) scores indicate overlap or unclear structure among clusters.

**FIGURE 9:**
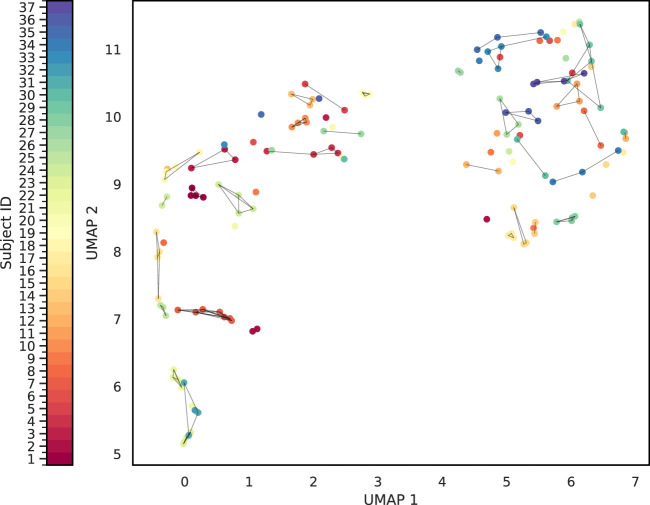
Cluster Analysis of the sample-level distance matrix. The UMAP representation of the sample level distance matrix. Points are connected based on whether belong to same sample and whether it is closed enough.

We quantitatively assessed clustering quality using the silhouette score, a well-established metric that compares each data point’s average distance to others in the same cluster against its average distance to points in different clusters. Overall, we obtained a mean silhouette score of 0.156, suggesting that, while some structure is present, the clusters are not strongly separated on average. To explore subject-level variations, we also plotted the distribution of silhouette scores for each subject (Figure 8). Approximately one-third of subjects exhibit well-separated clusters, another third show moderately acceptable clustering, and the remaining subjects have less well-defined structures. Notably, although the low-dimensional representation in Figure 9 shows that different time points from the same subject can appear spatially grouped, the clusters themselves are not well separated across subjects. This observation aligns with the slightly lower silhouette score, which reflects both intra-cluster cohesion and inter-cluster separation.

## Discussion

This study applied Quantized Optimal Transport (QOT) to analyze mass cytometry data from COVID-19 vaccinated cohort. Our approach uniquely avoids the biases of traditional gating by treating cell profiles as high-dimensional distributions. We demonstrated this method’s utility in identifying unstable proteins like CD3 and CD45, which varied significantly over time, indicating their active roles in the immune response to vaccination. Additionally, our study demonstrates the use of optimal protein combinations to find immune fingerprints for subjects. By using silhouette scores for clustering optimization, we identified protein sets that consistently group samples from the same individual across different time points, highlighting its potential for personalized medicine.

For future work, we aim to refine our analytical framework for high-dimensional mass cytometry data, enhancing its capability to handle large-scale datasets effectively. In our initial experiment, we employed an exclusion analysis to assess protein importance. Integrating methods such as Shapley values with Wasserstein distances could significantly enhance interpretability. Additionally, our current analysis does not account for subclusters within the distance matrices. Investigating these subclusters could reveal new phenotypic subtypes related to vaccination responses, providing insights into immune system dynamics.

## Data Availability

The original contributions presented in the study are included in the article/Supplementary Material, further inquiries can be directed to the corresponding author.
