# Supplementary material for: Optimal transport reveals immune perturbation and fingerprints over time in COVID-19 vaccination
Source: Exp Biol Med (Maywood). 2025 May 21;250:10445. doi: 10.3389/ebm.2025.10445 (PMC12135207; doi:10.3389/ebm.2025.10445)
Supplement: Supplementary file 1 [file DataSheet1.pdf]

# Supplementary

### Figure Legends for Appendix

Fig A.1. Leave-One-Out analysis for subject 6-16. Each line represents the perturbation in distance relative to the baseline at Day 0 over various time points. Different colored lines indicate the results using all proteins versus excluding a specific protein.

Fig A.2. Leave-One-Out analysis for subject 17-27. Each line represents the perturbation in distance relative to the baseline at Day 0 over various time points. Different colored lines indicate the results using all proteins versus excluding a specific protein.

Fig A.3. Leave-One-Out analysis for subject 28-37. Each line represents the perturbation in distance relative to the baseline at Day 0 over various time points. Different colored lines indicate the results using all proteins versus excluding a specific protein.

## Supplementary Tables

Table S.1: Cluster proportions for Subject 1. Each column corresponds to a cell type ID and shows its respective proportion of total cells.

| **Cluster ID** | 1 | 2 | 3 | 4 | 5 | 6 | 7 |
|---|---|---|---|---|---|---|---|
| **Proportion** | 0.10 | 0.15 | 0.20 | 0.15 | 0.20 | 0.10 | 0.10 |

Table S.2: Demographic details of Age

| **Age** | **Subjects** |
|---|---|
| 30-40 | 12 |
| 40-50 | 7 |
| 50-60 | 4 |
| Above 60 | 2 |

Table S.3: Demographic details of Gender

| **Gender** | **Subjects** |
|---|---|
| Female | 18 |
| Male | 19 |

Table S.4: Demographic details of Race

| **Race** | **Subjects** |
|---|---|
| American Indian or Alaska Native | 1 |
| Asian | 7 |
| Black | 3 |
| White | 24 |

Table S.5: Demographic details of Vaccination Type

| **Vaccination Type** | **Subjects** |
|---|---|
| Moderna | 4 |
| Pfizer | 33 |

## Supplementary Figures

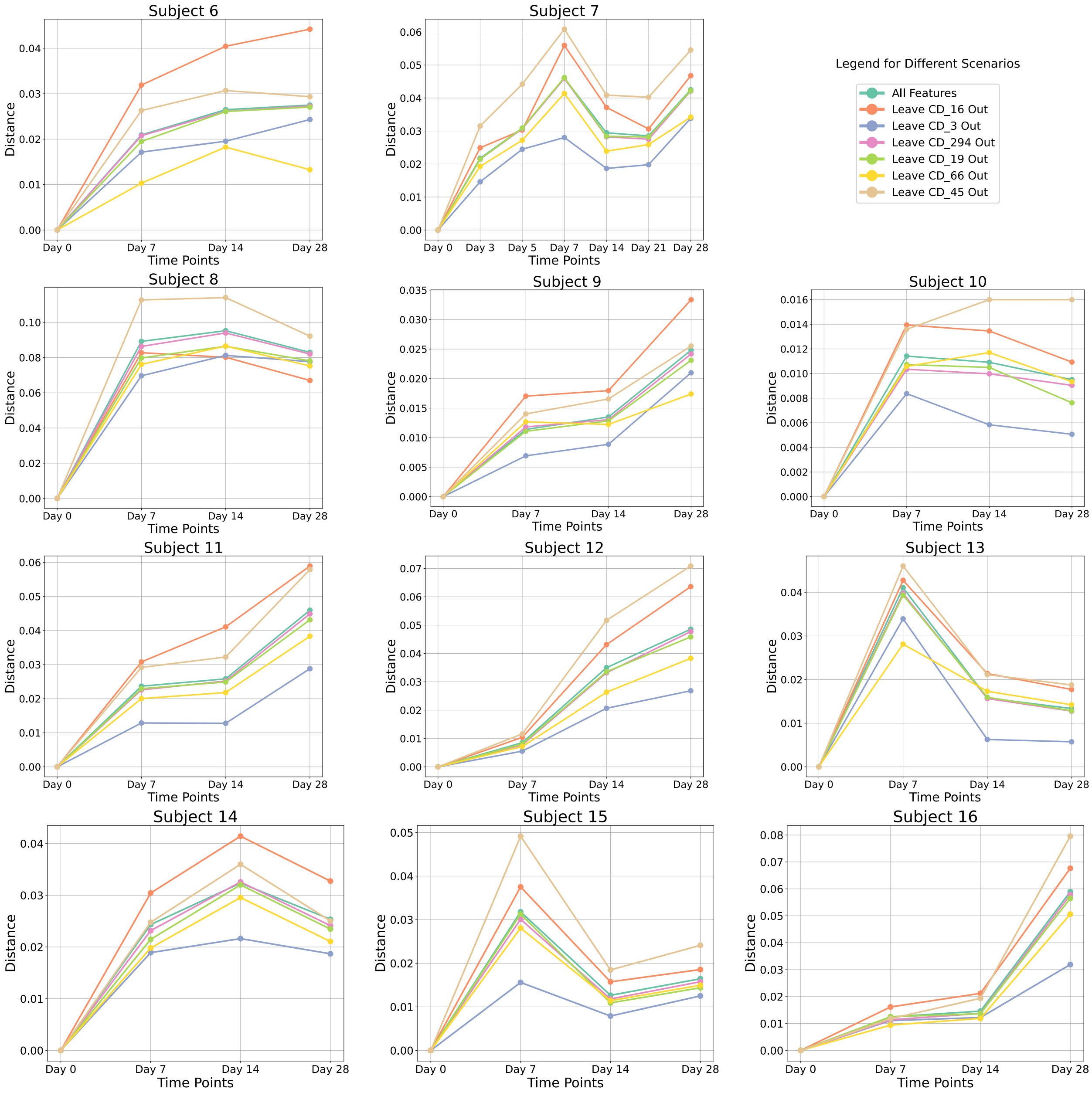


Figure A.1: Leave-One-Out analysis for subject 6-16. Each line represents the perturbation in distance relative to the baseline at Day 0 over various time points. Different colored lines indicate the results using all proteins versus excluding a specific protein.

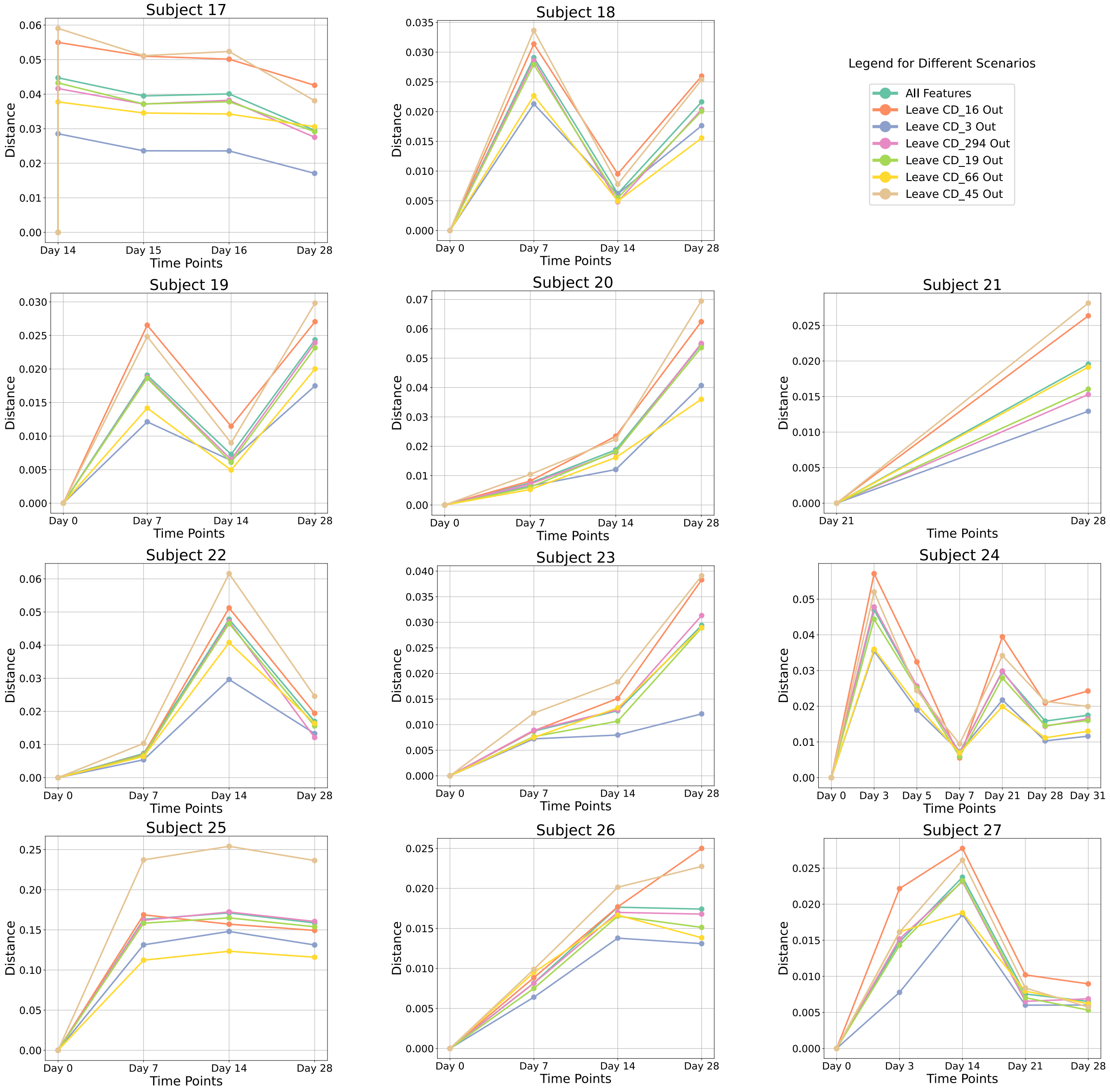


Figure A.2: Leave-One-Out analysis for subject 17-27. Each line represents the perturbation in distance relative to the baseline at Day 0 over various time points. Different colored lines indicate the results using all proteins versus excluding a specific protein.

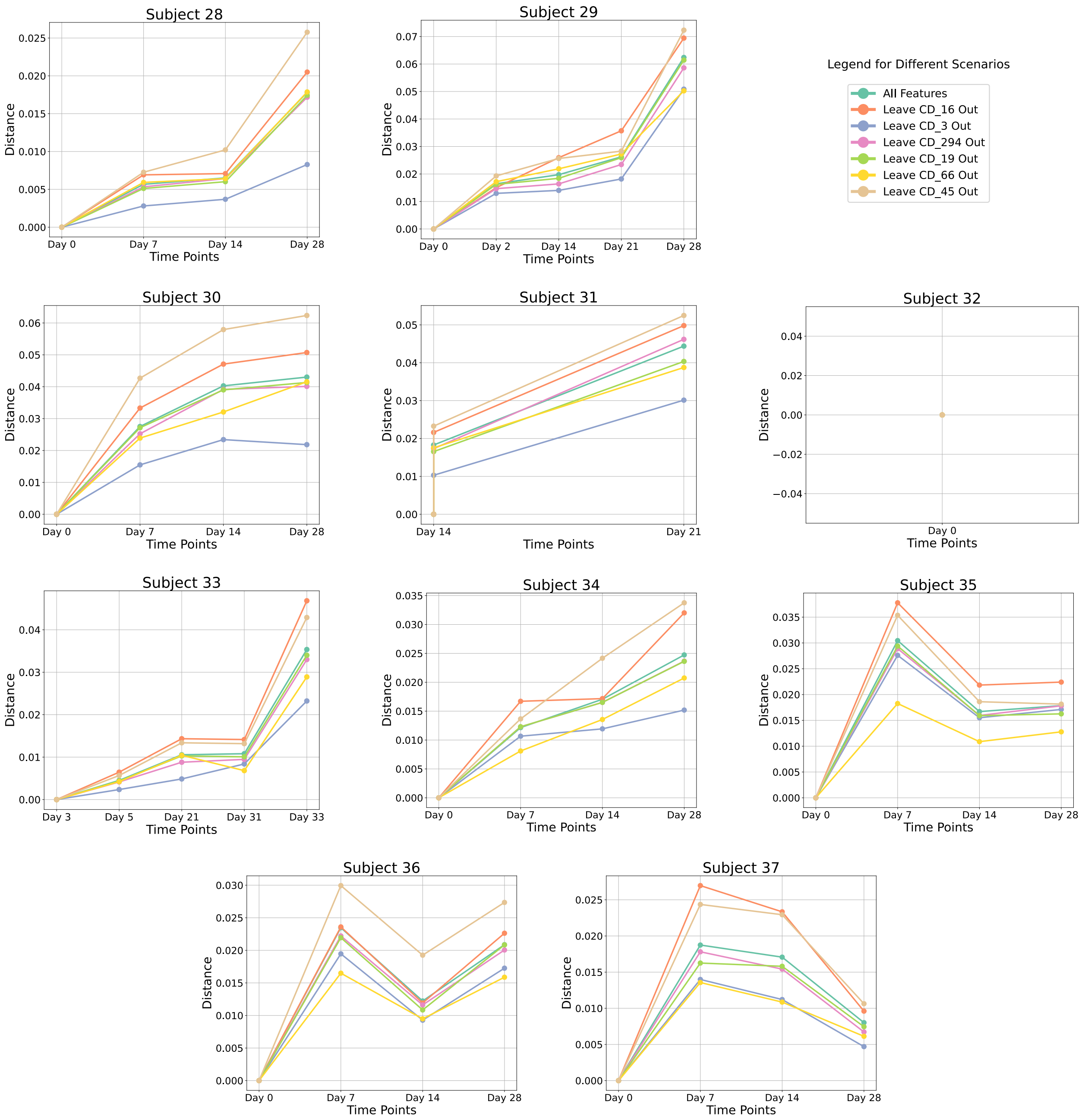


Figure A.3: Leave-One-Out analysis for subject 28-37. Each line represents the perturbation in distance relative to the baseline at Day 0 over various time points. Different colored lines indicate the results using all proteins versus excluding a specific protein.
